# “It’s Still Exposure Just in a Slightly Different Way”—Understanding the Contribution of Simulation to Developing Physical Therapist Skills in Ireland: An Interpretive Description Study

**DOI:** 10.1093/ptj/pzaf102

**Published:** 2025-08-15

**Authors:** Claire M Mulhall, Walter Eppich, Katharine Schulmann, Claire Condron, Suzanne McDonough, Orlagh O’Shea

**Affiliations:** RCSI SIM Centre for Simulation Education and Research, RCSI University of Medicine and Health Sciences, Dublin, Ireland; Society for Simulation in Healthcare Fellow, Faculty of Medicine, Dentistry and Health Sciences, The University of Melbourne, Melbourne, Australia; Centre for Health Policy and Management, Trinity College Dublin, Dublin, Ireland; RCSI SIM Centre for Simulation Education and Research, RCSI University of Medicine and Health Sciences, Dublin, Ireland; School of Physiotherapy, RCSI University of Medicine and Health Sciences, Dublin, Ireland; School of Physiotherapy, RCSI University of Medicine and Health Sciences, Dublin, Ireland

**Keywords:** Clinical Placement, Curriculum Design, Feedback Literacy, Physical Therapist, Simulation-based Education (SBE), Simulated Patient

## Abstract

**Importance:**

Simulation-based education (SBE) is increasingly used in physical therapist training to address growing student numbers and clinical placement shortages. However, clinical educators’ perspectives on the role of SBE in preparing students for practice remain unexplored.

**Objective:**

The objective of this study was to explore physical therapy clinical educators’ perspectives on academic-based SBE, particularly how it can equip students for clinical placement and whether it should contribute to practice education hours.

**Design:**

Qualitative interpretive description methodology using semi-structured interviews was used.

**Setting:**

Five hospital sites across the island of Ireland engaged in physical therapist practice education.

**Participants:**

This study involved 8 physical therapist practice educators and tutors with 6 to 15 years of experience, supervising 2 to 50 students annually.

**Intervention(s) or Exposure(s):**

Individual semistructured interviews were conducted exploring participants’ perspectives on SBE’s role in clinical education, lasting 40 to 60 minutes each.

**Main Outcome(s) and Measure(s):**

Thematic analysis identified patterns in clinical educators’ perceptions of SBE’s educational value and contribution to practice preparation.

**Results:**

Simulation supported the transition to practice by: (1) priming for clinical environments, (2) enhancing feedback literacy in the workplace, and (3) tackling complexity of clinical practice. Specific clinical skills including documentation, basic safety, manual handling, subjective assessment, and understanding the multidisciplinary team’s role were recognized as appropriate for instruction through SBE. Participants reported activities spent in SBE should count toward clinical hours and highlighted that processing feedback during SBE established a foundation for feedback practices in the workplace. Engaging simulated patients in scenarios informed by real patient experiences was proposed as a way of managing complex patient encounters.

**Conclusions and Relevance:**

SBE provides a means to scaffold the learning of essential clinical skills before practice placement and contributes to clinical education, though more research is needed to determine the proportion. Future research should examine simulated interventions to boost feedback literacy and readiness for clinical settings. Involving patients and the public in the design of SBE curricula is crucial for relevant and beneficial learning outcomes.

## INTRODUCTION

Simulation-based education (SBE) has emerged in recent years as a popular teaching modality in physical therapist training. Modes of simulation employed with varying levels of fidelity include part task trainers, human patient simulators (mannequins), virtual and augmented reality and engaging simulated participants.^1–4^ A recent scoping review by Stockert et al^5^ reported a 5-fold increase in citations on SBE use in physical therapist pre-registration education between 2012 and 2021 compared to preceding years. This increased use of SBE in physical therapist education is in response to the rising numbers of physical therapist students to meet the growing demand for physical therapists globally and the shortage of sites available for clinical placement.^6–8^

As part of physical therapist training, World Physiotherapy stipulates that all physical therapist programs dedicate one-third (or 1000 hours) of their program to practice education, defined as the delivery, assessment and evaluation of learning experiences in practice settings, including institutional, industrial, occupational, primary health care, and community settings.^9^ Typically, students meet the demands of practice education through immersive experiences in traditional clinical practice placements, where they are supervised by qualified physical therapists. However, workforce shortages and the burden and stress reported worldwide by clinical educators limit the clinical education learning opportunities available.^10–12^

The simulated learning environment links classroom and clinical settings, providing learners with opportunities to practice the skills essential for their professional roles.^13^ The learning theory that underpins SBE is experiential learning,^14^ which proposes that through repeated practical experience and feedback clinical competence and confidence can be developed. Evidence from undergraduate nursing programs indicates that substituting part of the required clinical practice hours with simulation is as effective as traditional clinical practice for developing confidence, skills, and knowledge.^15^^,^^16^ Similarly, undergraduate physical therapist SBE programs in Australia have been shown to meet clinical competency requirements, including professionalism and communication.^17^^,^^18^ Data suggest that 25% of clinical education can be replaced by simulated learning without compromising students’ attainment of professional competencies.^17^ Furthermore, the COVID-19 pandemic prompted a move away from traditional placement models toward more flexibility in how students achieve their 1000 hours of practice.^19^

Physical therapist governing bodies in Ireland offer little guidance regarding how many hours of simulation can be used to support the 1000 hours of workplace-based practice education. In contrast, the Nursing and Midwifery Council which prescribes standards for prelicensure nursing programs in the UK allow 600 hours of the required 2300 hours (26%) of practice education to be replaced with simulation practice.^20^ Moreover, in Australia Occupational Therapy accreditation standards endorse that SBE may replace up to 200 of the mandated 1000 practice-based learning hours (20%).^21^ Despite this lack of guidance, physical therapist educators globally have been routinely using SBE to support practice education.^22^ International educational processes need careful contextual consideration before adoption. For SBE to be formally accepted as able to contribute to physical therapist clinical education in Ireland, the perspective of clinicians, as those best placed to comment on the impact of the learner competency, must be sought.

While research has explored physical therapist academics’ perspectives of SBE,^19^^,^^23^ little if any research has explored how workplace-based clinical educators perceive SBE and its potential to better prepare students for the clinical environment. Arguably, better prepared students will be more self-sufficient and require less support in the clinical setting. Therefore, this study aims to explore physical therapist clinical educators’ perspectives of academic-based SBE, in particular how SBE can equip students for clinical placement and whether it should contribute to practice education hours.

## METHODS

### Study Design

We used a qualitative interpretive description (ID) methodology^24^ to explore participants’ perspectives. ID methodology is suitable for qualitative research across health professions when the goal is to explore and understand the subjective experiences of a population and use this knowledge to inform practice.^25^ While qualitative interpretation prioritizes theoretical contributions, qualitative ID emphasizes practicality, offering accessible and actionable findings. Thus, ID combines both descriptive and interpretive elements, aiming for clarity and relevance in real-world contexts.^26^^,^^27^ This approach suited this study as it offered a means to capture patterns and themes within subjective perceptions and experiences, while also acknowledging individual variations.^28^

### Setting and Recruitment

This research arose from a need identified by academic educators in another study.^19^ Uniquely, Irish physical therapist accreditation bodies are divided into 2 separate jurisdictions: the Republic of Ireland which is regulated by the Health and Social Care Professionals Regulator; and Northern Ireland which is regulated by the Health and Care Professions Council. Despite these separate governing bodies, practice education faces uniform issues.

Purposive sampling was used to identify 6 clinical sites engaged in physical therapist practice education located across the island of Ireland. Ensuring the perspectives of staff at clinical sites in different geographical areas, urban or less urban areas, and of different sizes and numbers of students facilitated per year are represented in the data. An invitation letter, participant information leaflet and consent form were sent via email to the physical therapist managers at each site seeking to recruit the clinical education leaders with responsibility for supervising students within each setting. The manager then distributed these documents and those interested in participating provided informed consent and online interviews were then scheduled.

### Data Collection

One investigator (O.OS.) conducted individual semi-structured interviews with 8 participants who gave their consent to partake in the study. The interview guide developed by the research team is included in the Supplementary Material. Simulation was defined using the Society for Simulation in Healthcare definition^29^ at the outset of the interviews. All interviews were conducted between October 2021 and February 2022 via video conferencing technology Microsoft Teams (Microsoft Corp, Redmond, WA, USA), audio recorded, transcribed verbatim, checked for accuracy and de-identified. The interviews ranged from 40 to 60 minutes in duration. Demographic data was collected including: sex; years of experience as a physical therapist educator; whether they were a practice educators or tutors and approximately how many students they supervise at their clinical site per year. The first and second interviews were discussed between the first and senior author to assess the interview guide, which resulted in minor guide adjustments for the following interviews.

### Reflexivity

Throughout the study, the authors reflected via discussion on how their individual perspectives shaped the research process.^30^ The authors have expertise in health professions educational research, physical therapist education, SBE and sociology. All authors have experience with qualitative research methods. Three authors (C.M., W.E., C.C.) are experienced simulation educators. The senior author (O.OS.) is an academic physical therapist educator and another author (S.McD.) is the Head of a School of Physiotherapy; neither is directly involved in practice education. To ensure an external perspective and to provide a distinct lens, 1 author (K.S.) is a researcher with a background in health and social care systems research.

### Data Analysis

Prior to analysis, the transcripts were made available to the participants for review and clarification. Thematic analysis was used to analyse the interview transcripts.^31^^,^^32^ As ID does not prescribe a singular technique for data analysis, we followed the process laid out by Braun and Clarke,^31^ by (1) familiarising ourselves with the data, (2) generating initial codes, (3) searching for themes, (4) reviewing themes, (5) defining and naming themes, and (6) producing the report.

During initial analysis, early interviews were independently coded manually line-by-line by 3 authors (C.M., K.S., O.OS.). We employed constant comparison to generate codes. Consistent with ID, knowledge gained from early interviews guided subsequent data collection and analysis. As our initial coding scheme matured, C.M. and O.OS. coded the remaining interviews, meeting frequently to refine the codebook through discussion to achieve consensus. These iterative discussions resulted in combining and sorting codes into candidate themes and sub-themes. These were refined into overall themes and representative quotes from the interviews were selected to illustrate them. Relevant quotes were debated, discussed, and eventually agreed upon. Transcripts were revisited to ensure no important information was overlooked or misinterpreted during the thematic analysis and to clarify the relationships between the themes. Data collection was concluded by assessing the data adequacy, which is comprised by the theoretical sufficiency, information power, and conceptual depth.^33–35^ This was discussed as a team at the outset and throughout the active phase of data collection. Discussion focused on whether there was adequate variation and complexity, and whether the data were rich and relevant enough to offer meaningful insights to guide practice.^36^ Microsoft Word (Microsoft Corp, Redmond, WA, USA) was used for data management, coding, and memo writing.

Rigor and trustworthiness in this study were addressed through several methods, including a structured study design, systematic yet flexible sampling approach and ongoing consultation between the research team. Participants were able to speak freely during interviews and precise transcription allowed for strong positioning of participants’ experience and voice through use of quotes when presenting each theme. Constant comparison maintained focus on content and facilitated data-driven coding. To transition from the descriptive to interpretive phase, the authors consistently posed questions such as: What am I observing? Does this surprise me, and why? What are the implications and recommendations for simulation-based physical therapist education?^37^ Our processes were informed by the Standards for Reporting Qualitative Research.^38^

### Role of the Funding Source

The funder played no role in the design, conduct, or reporting of this study.

## RESULTS

Eight semistructured interviews with physical therapist practice educators and tutors involved in leading clinical education activities at 5 hospital sites located across the island of Ireland were completed (1 site invited to participate never responded). The Table provides an overview of relevant participant characteristics.

**Table:** Participant Demographics

**Participant ID**	**Sex**	**Years of Experience**	**Role**	**No. of Students Facilitated per Yr**	**Geographic Location**
P01	Female	10	Practice educator^*a*^	3	South
P02	Female	9	Practice educator	2	West
P03	Female	8	Practice tutor^*b*^	28	West
P04	Female	7	Practice tutor	20	Midlands
P05	Female	15	Practice tutor	50	North
P06	Female	8	Practice educator	2	East
P07	Female	6	Practice tutor	10	East
P08	Female	7	Practice tutor	15	East

a Individual therapists who educate, monitor, and mentor students when on placement.

b Posts funded by the public health service at senior therapist grade to develop the capacity and quality of the practice education program and support both students and practice educators.

Our analysis revealed that simulation supported the transition to practice in 3 ways: (1) priming for clinical environments, (2) enhancing feedback literacy in the workplace, and (3) tackling complexity of clinical practice. These themes delineated 3 critical stages of learning where simulation can assist in fostering students’ placement education preparedness through an integrated and scaffolded approach. We now consider each theme and identify representative quotations using anonymous participant codes (eg, P03).

### Priming for Clinical Environments

Participants placed high importance on building students’ confidence by practicing skills in advance of clinical placement. The overwhelming sensory nature of clinical environments emerged as a challenge that participants believed academic-based SBE could help mitigate through gradual exposure.

“All the alarms, all the beeping, [the] smell. To be in ICU and you’re suctioning and there’s the noise and there’s the texture, and they’re like, oh my God.” (P02).

This vivid description underscores how environmental factors may hinder learning, indicating that simulation provides essential desensitization prior to actual clinical experience.

Many discussed how specific clinical skills and competencies could be better developed through SBE prior to placement and how “having a bit more practice straight before their placement...having it in the forefront of your mind what the skills for that placement are going to be” (P08) would be valuable. This included students’ documentation skills and brevity when relaying their assessment.

“[I tell them] I need you to come and find me and tell me in 1 sentence what happened today. What’s the sentence? ... Whatever the assessment is, it’s like they either keep it too vague with treatment went well, or it’s an essay.” (P01).

This frustration highlights a gap between academic preparation and clinical expectations, suggesting that SBE could provide more structured practice in documenting patient assessments.

Participants discussed the tension between the expectation to learn skills on-site and the practical limitations imposed by time pressures in busy clinical settings. Using SBE to practice “basic safety” (P03), “manual handling equipment” (P06) and understand “what the MDT [multidisciplinary team] do” were identified as beneficial as “educators don’t really have the time to be going back to basics...how to put the brakes on the bed and all that kind of stuff” (P03).

Priming through SBE extended to students’ history-taking skills and their ability to structure and practice these skills prior to interviewing real patients.

“I suppose in terms of communicat[ing] with patients, … role-plays might be an idea so that they can have a structure on how they’re asking their subjective assessment. Just so they know the specific questions…to be asked” (P03).

Some lamented that students don’t get enough objective skills practice at university prior to their clinical placement and are relying on clinical sites to provide this repeated practice.

“I think they don’t get enough practice at the moment...a huge amount of emphasis goes into theory and research...but the practical skills they’re hoping that it all happens on site, but they do need some elements of preparation in advance” (P02).

Others outlined how simulation can better prepare students to have “confidence to speak to other members of the team and get information that way” (P04) and conduct specific patient assessments while on placement.

“For practicing… a respiratory assessment, on a patient. They could practice on each other or a mannequin, an actor or something, to practice their actual respiratory assessment skills before going on placement” (P04).

While participants shared the sentiment that “you can’t beat real-life scenarios, real-life patients” (P02), many supported simulated activities contributing towards practice placement hours. COVID-19 justified this shift. When clinical placements became limited, simulated activities usefully contributed to clinical placement hours.

“Just because it’s a simulated scenario over a real-life scenario doesn’t mean that it shouldn’t count. Sometimes they might come on placement, and there might be things they won’t see, and they’re not going to see everything...say during COVID. It’s not going to be the same as a clinical situation, but I think it’s got a huge amount to add that’s very similar. [Simulation] would be of benefit to count towards clinical hours because it’s still exposure just in a slightly different way” (P08).

Responses varied in terms of the proportion of clinical training hours via simulation that would be acceptable. However, most participants indicated that the “bulk of their hours should be in physically [the clinical environment]” (P03); 1 participant highlighted that the educational experience itself was paramount, and not an arbitrary number of hours.

“It depends on the actual experience you have, even the 1000 hours, you could get the same experience in 200 hours depending on the amount of [operating] theatre time they give you, the amount of hours is probably quite arbitrary” (P06).

### Enhancing Feedback Literacy in the Workplace

Simulation offered students the opportunity to repeatedly practice scenarios and receive guidance while building confidence and minimizing nervousness. Clinical educators described how participating in simulation at university prepared students to maximize learning opportunities during clinical placement.

“They’d benefit more from the placement if they’d already gone through a scenario… and I think it would help them have better outcomes on the placements” (P02).

Learning to process feedback during SBE also set the scene for feedback practices during clinical placements. Multiple participants described how they approached giving feedback to students mirroring the process in SBE by supporting them to analyse their thoughts and actions via regular feedback and debriefing sessions offering opportunities for reflection, learning and growth.

“It’s constantly teasing out how are they thinking. How are they clinically reasoning through something?... There would be very much an informal [approach], like, “Hey, can I just get you to check X or Y,“ and you come over. But there would usually be, nearly daily feedback and debriefing on how things went” (P01).

Others discussed how they moderated their approach over the duration of the placement to help develop students’ critical thinking skills.

“You start off giving by them specific feedback. Then as the weeks go on, it’s important to get them to reflect” (P03).

However, participants identified a paradox in which patterns of academic success became an obstacle to clinical learning when students had difficulty discussing their learning through feedback conversations. When asked how prepared learners were for feedback during their placements, participants shared challenges experienced in encouraging students to understand, process and use feedback to enhance their learning.

“The very high-achieving person who was quite anxious and they’re so super anxious to do well that any feedback that’s constructive is seen as nearly catastrophizing. It’s my job to give feedback. And if it’s taken on board and internalized and acted on, we’re golden. But [it’s problematic] if somebody can’t hear you because they’re too overwhelmed or they’re just defensive.” (P01).

This paradox indicates that simulation may be vital in reframing feedback as a learning tool to enable students to self-evaluate, particularly for high-achieving students.

Participants discussed the benefits of role-play in advance of clinical placement and how these types of simulated scenarios can be developed to help familiarize students with feedback practices and normalize the process of discussing instances when aspects don’t go well during clinical placement.

“Peer-based training is really valuable and those role-playing techniques. In practice when you’re learning, to make things go wrong and have role-plays where everything is just not going to go right and to be able to reflect on that and why. I think that’s just as valuable as having a role-play where you get all your assessments done” (P06).

### Tackling Complexity of Clinical Practice

As students progressed through their academic programs, they encountered patients with increasingly complex needs and challenges during their clinical placements. Participants emphasized the importance of aligning students’ pre-placement training with this escalating complexity to help them learn how to adjust their approaches during placement based on individual patient needs and changing circumstances.

“Sometimes [with] the more complex, the more dense strokes, often, students nearly get a bit of a fright. They don’t quite expect that you have to give a large amount of assistance to help move the leg or, for example, that you have to support the arm fully. So, just to have a good understanding and awareness of that before they come” (P07).

The importance of incorporating diverse and complex cases, based on real patient experiences, to enhance students’ ability to critically analyse situations, explore different perspectives, and develop innovative solutions while on clinical placement was highlighted as “it doesn’t become real until you’re faced with somebody, and they have 15 steps before they get in the front door” (P01).

Participants acknowledged that effective clinical practice requires interpersonal skills, which can only be cultivated through experience with challenging scenarios. They emphasized the value of preparing for handling situations while on placement that require a blend of patience, understanding, and effective communication strategies.

“If a patient is declining a discharge to a certain destination, for example, and maybe it’s the only option for them and for their age group, so you’re trying to bring them around to see the benefits of 1 new location over the other” (P07).

The limitations of idealized academic scenarios became apparent when students faced the unpredictability and complexity of real patient interactions. Participants shared helpful insights about explicit teaching interactions using simulation that could inform how we prepare students to navigate challenging situations effectively, ensuring better outcomes and maintaining positive interactions, even in demanding circumstances.

“It’s very difficult to prepare for those kinds of scenarios when you’re in the college. You learn how the perfect scenario would go, and then you come to see patients and they’re delirious, or they’re getting quite aggressive and [students] are learning pieces that would have benefited from simulation” (P06).

Participants were keen to highlight that “when we learn these things if we learn a perfect way of how it would go, in practice it doesn’t always work that way” (P06) and that navigating patient interactions involves dealing with unpredictability since every patient interaction brings an element of uncertainty. Participants described how simulation could prepare students for the uncertain nature of clinical environments.

“You’re dealing with a patient, you don’t know really what the response is going to be, it’s unpredictable, the workplace is unpredictable, patients are unpredictable and it’s really important that any simulation keeps up” (P05).

This focus on unpredictability frames simulation not just as rehearsal for specific situations, but as practice for adapting communication approaches in real-time to meet a variety of patient needs.

Many suggested that “using role play and actors [was] probably a good way to go” (P07) to provide opportunities for students refine their communication skills, especially when dealing with patients who present with complex issues such as “somebody who is slightly deaf, recovering from a delirium, has a mild dementia” (P01).

The potential for simulation to replicate realistic multi-morbidity scenarios reflecting current health care challenges was considered especially valuable. Multiple participants described how actors or simulated patients might help students to practice realistic scenarios that mirror the complexities of patient care in a controlled and safe educational environment.

“Particularly the elective patients [who] aren’t presenting with just 1 problem. There’s multiple problems... Maybe they could write a script based around those types of patients... If the simulation was a person as opposed to a machine, you could possibly do that. You would write a script to have lots of alternatives and things thrown up for the student” (P02).

In situations where patients might resist or decline recommended treatment or display behaviors that impede effective communication, participants considered that scenarios involving simulated patients presented a valuable tool for students.

“Not every patient is...compliant and willing to work with you. Sometimes, there are those conversations that have to happen if a patient is declining physio, for example, and you know they really need to be seen or a patient who maybe gets difficult with you. So, I think there is a role for role play as well… in those more complex situations” (P07).

The value of patient perspectives in the learning process was highlighted. Participants acknowledged that simulated patient feedback offered unique insights not accessible through other assessment methods, emphasizing that “patient feedback is the best thing” (P07).

## DISCUSSION

Our findings captured the varied experiences of clinical educators responsible for supervising physical therapists-in-training in Ireland and their perspectives on the role of SBE in clinical education. Participants identified specific clinical skills which should be taught via SBE to help students prepare for the clinical environment. They discussed the potential contribution of SBE to practice education hours. They highlighted the possible contribution of SBE in fostering feedback literacy. Finally, participants identified the key role SBE, and simulated patients in particular, can play in preparing students for navigating complex patient encounters. We now expand on these points.

### SBE as a Scaffolded Learning Environment

The clinical skills identified by participants serve as an initial step towards acceptance that SBE can contribute to physical therapist clinical education in Ireland. These skills align with the identified core characteristics which demonstrate students’ readiness for the clinical environment.^39^^,^^40^ To learn these prerequisite skills, SBE offers the perhaps ideal scaffolded learning environment to gain these skills without patient risk. Moreover, participants highlighted that participating in SBE directly prior to placement would be beneficial as these skills would be at the forefront of students’ minds. While the use of SBE before clinical placement has been explored in the literature with positive outcomes for students,^41^ we contend that the optimal timing of SBE will vary depending on the local curriculum and learners’ needs.

### Contribution to Practice Education Hours

The majority of participants in the current study agreed that activities spent in SBE should count towards clinical hours, signifying a new shift in clinician support for SBE contributing towards practice placement hours. The arbitrary nature of the 1000 hours required underscores the lack of standardization in practice placements causing inconsistencies across sites, encouraging an emphasis for students on grades rather than on learning.^42^ SBE therefore represents a way to standardize the student experience allowing clinical competence to demonstrated, particularly during exceptional situations like the COVID-19 pandemic.

Medical education has shifted from time-based clinical education to competency-based education.^43^ Entrustable professional activities (EPAs), first introduced by ten Cate,^44^ represent the most widely adopted assessment framework, anchoring competencies in various everyday skills.^45^ Some initial work has been done on the development of EPAs in physical therapist education.^46^ We consider that an EPA-based physical therapist curriculum, underpinned by SBE, would offer a safe and standardized way to progressively increase learners’ responsibilities and autonomy while in the clinical environment;^47^ resulting in increased patient safety and a reduction in the burden and stress reported by clinical educators. Moreover, the specific clinical skills identified by participants as appropriate for SBE represent core units of activity expected to be performed by entry-level physical therapists and as such could form part of an EPA-competency matrix. We consider that as simulation allows for direct observation, assessment tools for evaluating EPA performance in simulation could potentially offer supplementary data to complement clinical assessments.^48^

### Feedback Literacy

Participants identified feedback, debriefing and reflection as strategies they used to help students both acclimate to the clinical environment and develop their clinical skills as they progress. Feedback literacy is defined as “the understandings, capacities and dispositions needed to make sense of information and use it to enhance work or learning strategies” (p1316).^49^ It has been identified as an important concept in health care to ensuring high-quality learning experiences, enhancing professional and clinical competency and developing lifelong skills for reflective practice; ultimately improving patient care.^50^ Developing learners’ feedback literacy and evaluative judgement of their clinical practice can help to build competence and autonomy.^51^ Participants described how the feedback process can become problematic when students have negative emotional responses to feedback information. Responding to feedback without defensiveness is a characteristic of readiness for clinical placement^39^^,^^40^ and an important domain of feedback literacy.^52^

SBE has the potential to offer a feedback-rich experience since, during a simulation, the primary focus is on student learning rather than patient care. This means that time for feedback can be planned and prioritized.^13^ Furthermore, simulation can potentially offer a more enhanced feedback experience than the clinical environment as students can receive feedback from simulated patients; acting as proxies for real patients.^53^ Student encounters can be video recorded to enable self-assessment and reflection. SBE has been used also as a means of developing students feedback skills through peer assessment^54^^,^^55^ and peer observation^56^ which are not always possible in the clinical environment. To our knowledge no study has explored the role of simulation in improving physical therapist student feedback literacy and based on the views of participants in this study we believe this exploration would be welcomed. Future research should explore simulated interventions to enhance feedback literacy and its impact on students’ preparedness for the clinical environment.

### Navigating Complex Patient Encounters

As students’ progress in their training, they gain exposure to patients with increasingly more complex clinical, behavioral and social presentations. Notwithstanding, their perspective that real-life scenarios with actual patients are unrivalled, participants highlighted the importance of using simulation to prepare students for complex patient encounters and the unpredictability of the clinical environment. Moreover, although clinical placements are a vital part of the learning process, students working with patients with conditions such as dementia have reported how their pre-placement training left them feeling unprepared and consequently recommended SBE as part of their future training.^57^

An integral part of students’ simulation-based communication skills training involves working with simulated participants (SPs) who facilitate active participation in learning activities encouraging constructive and supportive learning conversations.^58^ We define a SP as a person who portrays a patient (simulated patient), family member, or health care professional to meet the objectives of the simulation. Physical therapist students who engaged with SPs during their training have reported consistently that learning with SPs felt more “real” than role play and therefore more valuable to them.^2^ SPs have been engaged to prepare students for the development of communication skills^59^ and acquisition of basic patient-centred skills.^60^ Their involvement has broadened to include preparation for the complexities of clinical practice including navigating ethical issues typical in practice^61^ and challenging conversations.^62^ SPs are educators in health professions education, trained to adapt and tailor their role portrayal and feedback practices in response to learners’ educational needs.^63^ This vital educative role has been shown to have satisfied clinical competency requirements, including those associated with professionalism and communication, in simulated learning programs related to musculoskeletal,^17^ and cardiorespiratory^18^ physical therapist practice in Australia. Participants identified specific scenarios suitable for SP engagement including cases relating to communication barriers and patients resisting or declining treatment. We consider engagement with practice educators and tutors in the design of SP scenarios important in ensuring their clinical relevance.

The importance of including a diversity of cases, drawn from real patient experiences, was emphasized to help students prepare for the demands of the clinical environment. We consider that the development of an integrated SBE curriculum using SP methodology^64^ would facilitate this. We recommend that physical therapist educators experienced in SBE use participatory design^65^ to collaborate with interested parties, including clinicians, students, SPs and patients to design and develop SP scenarios tailored to the learners and context. We believe that incorporating patient and public involvement (PPI) in the design of SBE curricula is crucial for ensuring learning outcomes are both relevant and beneficial to the individuals who will ultimately be impacted by the knowledge and skills of physical therapist graduates. Patients and the public have an integral role in educating health care professionals, and their involvement is considered essential for high-quality education.^66^ Their input can help educators create meaningful and realistic simulated scenarios that reflect the complexity and diversity of actual patient care.

### Limitations

This research has important limitations. Our participants self-selected to participate. Participant diversity was limited in terms of sex mirroring the composition of the physical therapist work force globally. Given the small sample size the findings and lessons gleaned may not be applicable to all contexts. One clinical site did not respond to the invitation to participate and so the perspective of clinical educators at that site was not captured. Another limitation is the timing of data collection which took place during the COVID-19 pandemic. This likely had an influence on the views of study participants about SBE. Our study was designed to focus on academic-based SBE and so it is not definitive to what extent these results would be representative of clinical educators’ views of in-situ SBE.

## CONCLUSION

SBE is growing in popularity in physical therapist education. It offers a scaffolded learning environment to prepare undergraduate students for key transitions in their placement education. SBE provides a means of preparing students for the clinical environment during their training in key areas, including manual handling, safety, assessment skills and managing complex patient encounters. Simulated practice learning can contribute to practice learning hours; further work is needed to determine the appropriate proportion. Our findings show that, although not a substitute for exposure to the in-person, clinical environment, SBE provides students with a strong educational foundation and helps cultivate their abilities as reflective practitioners. PPI in the design of SBE curricula is necessary to improve teaching and learning by better representing the patient population and ensuring that SBE mirrors the complexities of clinical practice.

## Supplementary Material

2024-0648_R2_Supplementary_Material_pzaf102

## Data Availability

The data supporting the findings of this study are available from the corresponding author upon reasonable request.
