# Supplementary material for: “It’s Still Exposure Just in a Slightly Different Way”—Understanding the Contribution of Simulation to Developing Physical Therapist Skills in Ireland: An Interpretive Description Study
Source: Phys Ther. 2025 Aug 15;105(9):pzaf102. doi: 10.1093/ptj/pzaf102 (PMC12463445; doi:10.1093/ptj/pzaf102)
Supplement: 2024-0648_R2_Supplementary_Material_pzaf102 [file 2024-0648_r2_supplementary_material_pzaf102.pdf]

Supplementary Material

Interview guide

Opening of the Interview	
- Clarify purpose of the interview<br>- Confirm permission to audio record the interview	
Probes	
- Can you please tell me more?<br>- Could you give me an example of that?<br>- Can you give me an instance of that?<br>- How do you go about that?<br>- What makes that challenging?	
Topic	Questions
Topic One: Experience of simulation-based education	What experience do you have of simulation-based education either as a learner or as an educator?
Topic Two: Current teaching practices for practice education	What activities do you as an educator use to enhance student preparation or performance in the clinical environment? (prompts - recommending pre-placement reading, any engagement with university lecturers, tutorials or activities that involve patient contact)
Topic Three: Potential role for SBE in physiotherapy education	1. What, if any, specific skills could or should students be better prepared for when entering the clinical environment? (prompts - in relation to for example communication, patient assessment or treatment, professionalism or documentation)<br>2. What thoughts do you have about the use of simulation-based education [provide example from answer to previous question] to practice these skills? (probe barriers, facilitators, timing (pre-placement/during placement), setting (university, hospital site))
Topic Four: Potential role for SBE in physiotherapy practice education	One area we are examining is whether these purposive and structured simulated activities to prepare students for the clinical environment should count towards practice placement hours. What are your thoughts on this? [Prompts if negative, why? If positive, why? Is there a certain amount of hours that would be acceptable?]
